# Mutational spectrum in patients with dominant non-syndromic hearing loss in Austria

**DOI:** 10.1007/s00405-024-08492-5

**Published:** 2024-02-24

**Authors:** Alexandra Frohne, Sybille Vrabel, Franco Laccone, Juergen Neesen, Sebastian Roesch, Silvia Dossena, Christian Schoefer, Klemens Frei, Thomas Parzefall

**Affiliations:** 1 Department of Otorhinolaryngology, Head and Neck Surgery, Medical University of Vienna, Waehringer Guertel 18-20, 1090 Vienna, Austria; 2 Department for Cell and Developmental Biology, Center of Anatomy and Cell Biology, Medical University of Vienna, Vienna, Austria; 3 Institute of Medical Genetics, Center for Pathobiochemistry and Genetics, Medical University of Vienna, Vienna, Austria; 4 Department of Otorhinolaryngology, Head and Neck Surgery, Paracelsus Medical University, Salzburg, Austria; 5 Institute of Pharmacology and Toxicology, Paracelsus Medical University, Salzburg, Austria; 6 Present Address: Danube Private University, Steiner Landstraße 124, 3500 Krems a.d. Donau, Austria

**Keywords:** Autosomal dominant hearing loss, Syndromic hearing loss, ADHL, Variant interpretation, Mutational spectrum

## Abstract

**Purpose:**

Hearing loss (HL) is often monogenic. The clinical importance of genetic testing in HL may further increase when gene therapy products become available. Diagnoses are, however, complicated by a high genetic and allelic heterogeneity, particularly of autosomal dominant (AD) HL. This work aimed to characterize the mutational spectrum of AD HL in Austria.

**Methods:**

In an ongoing prospective study, 27 consecutive index patients clinically diagnosed with non-syndromic AD HL, including 18 previously unpublished cases, were analyzed using whole-exome sequencing (WES) and gene panels. Novel variants were characterized using literature and bioinformatic means. Two additional Austrian medical centers provided AD HL mutational data obtained with in-house pipelines. Other Austrian cases of AD HL were gathered from literature.

**Results:**

The solve rate (variants graded as likely pathogenic (LP) or pathogenic (P)) within our cohort amounted to 59.26% (16/27). *MYO6* variants were the most common cause. One third of LP/P variants were truncating variants in haploinsufficiency genes. Ten novel variants in HL genes were identified, including six graded as LP or P. In one cohort case and one external case, the analysis uncovered previously unrecognized syndromic presentations.

**Conclusion:**

More than half of AD HL cases analyzed at our center were solved with WES. Our data demonstrate the importance of genetic testing, especially for the diagnosis of syndromic presentations, enhance the molecular knowledge of genetic HL, and support other laboratories in the interpretation of variants.

**Supplementary Information:**

The online version contains supplementary material available at 10.1007/s00405-024-08492-5.

## Introduction

Hearing loss (HL) is among the most common disabilities and affects more than five percent of the world population (430 million people) to a significant degree [1]. The etiologies are diverse, but in many cases, the cause is genetic. Variants in more than 120 genes have been linked to non-syndromic HL following Mendelian inheritance patterns [2], and the list of ‘deafness genes’ is still expanding. The clinical presentation typically correlates with the mode of inheritance. Whereas most cases of autosomal recessive (AR) HL are congenital or manifest in early childhood, dominant (AD) HL is typically associated with a later onset and often progressive. Genetic diagnoses are critical for genetic counseling, family planning, and therapy choices [3]. This is particularly important for syndromic cases that might be misdiagnosed as non-syndromic if additional symptoms have been subclinical, have not developed yet, or if the link to the auditory phenotype has not been recognized. The identification of involved genes and variants continues to improve genetic diagnoses and provides insights into molecular inner-ear function and disease. Adding to the current significance, genetic HL has attracted interest as a candidate for treatment with gene therapy [4–6].

Genetic diagnoses are complicated by the genetic heterogeneity of the disease. Only two genes, *GJB2* and *STRC*, have emerged that, in some populations, account for double-digit percentages of diagnoses in congenital severe-to-profound AR HL (50% [7, 8]) and early-onset moderate AR HL (up to 30% [9]), respectively. In AD HL, no gene accounting for comparable case numbers has been identified, and many causative variants are private to isolated families. Moreover, the pathogenicity assessment of novel variants is commonly hampered by a lacking availability of functional assays or family members for segregation analysis [10]. Efforts to standardize the interpretation of sequence variants [10–12], the continued publication of novel disease and candidate variants, and the deposition of genetic and patient data in public databases aim to improve consistent variant evaluations across laboratories and institutions [13, 14]. The growing pool of publicly available data further improves diagnosis by enabling the identification of mutational profiles and genotype–phenotype correlations of individual genes [10, 11, 15].

In a prospective genetic study, we have analyzed 27 Austrian families with clinically suspected non-syndromic AD HL. Here, we present and recapitulate the results from these efforts, including 18 unpublished cases screened by whole-exome sequencing (WES) and targeted analysis of AD HL genes. In about three quarters of the cohort cases (20/27), candidate causative variants could be identified, including 16 variants graded as likely pathogenic (LP) or pathogenic (P). Ten variants were novel (six graded as LP/P and four variants of unknown significance (VUS)). Novel missense variants were validated and characterized using literature research and prediction tools, and possible effects on the gene products were assessed using protein structures or models. Additional mutational data were gathered from two Austrian diagnostic centers and from literature to provide an overview of the Austrian mutational AD HL spectrum. Taken together, these results enhance the current understanding of AD HL genetic variation, contribute to an improved assessment of novel variants in the future, and provide insights into the genetic profile of AD HL in Austria.

## Materials and methods

### Clinical testing and patient selection

Patients from 27 families with clinically suspected non-syndromic AD HL were recruited as part of the ongoing prospective genetic study. Affected study participants underwent a clinical audiologic examination and pure-tone audiometry. Medical and family histories were taken, including the age of onset, exposure to ototoxic substances or noise, and present or past additional audiologic complaints. Inclusion criteria were the presence of HL, a positive HL family anamnesis, and a presumed AD mode of inheritance as inferred from the pedigree. Ethical approval of the study protocol was received from the ethics committee of the Medical University of Vienna (approval number: ECS 198/2004; annual extensions to date). All patients and parents of minors gave informed consent for participation in the study and the publication of genetic and clinical data.

### Targeted genetic analysis

Blood samples were taken and chromosomal DNA was extracted from fresh or frozen blood using a commercial isolation kit. The workflow of previously published cases is presented in detail in the respective publications [3, 15–17]. Panel sequencing of the previously unpublished cohort cases AD-7 and AD-27 was performed externally as part of a collaboration, as described in the supplementary file (Table S1). For the remaining index patients first presented in this study, WES on a NovaSeq 6000 device (Illumina, San Diego, CA, USA) and targeted analysis of all AD HL genes known at the time of the analysis were performed using a virtual gene panel (specified in Supplementary Table S2). Rare (≤ 0.001 in gnomAD v2.1, by reference to [10]) non-synonymous exonic and splice-site variants were validated by PCR and subsequent Sanger sequencing. In patients without exonic/splice-site variants graded as LP or P, the effect of all rare (by reference to gnomAD v3) synonymous or intronic variants on splicing within the covered regions of AD HL genes was assessed using SpliceAI [18]. In six of these families, additional family members could be recruited for parallel exome analysis or segregation analysis by PCR and Sanger sequencing. The pathogenicity of all novel variants was assessed by reference to the ClinGen-defined HL-specific recommendations for application of the ACMG/AMP criteria [10–12].

### Protein alignments and models

Alignments of peptide sequences retrieved from UniProt or Ensembl were performed to assess the conservation levels of residues affected by novel candidate variants using Clustal Omega [19] or COBALT [16, 20]. In the present study, ACTG1, PTPRQ, WFS1, and MITF peptide sequences of species from multiple mammalian and non-mammalian classes were aligned. For the mammalian proteins NLRP3 and CEACAM16, representative peptide sequences were selected from major mammalian lineages. All species and accession numbers are specified in Supplementary Table S3. Protein structures of NLRP3 (6NPY) and ACTG1 5JLH) from the RCSB PDB database (rcsb.org/) and the human CEACAM16 (AF-Q2WEN9-F1), WFS1 (AF-O76024-F1), and MITF (AF-O75030-F1) AlphaFold models were used to illustrate the location of the respective variant using PyMOL or Jmol (jmol.org/) [15, 16, 21, 22]. Mutant residues were introduced with MISSENSE3D [23] and visualized in PyMOL (Schrodinger, LLC. 2010. Molecular Graphics System, Version 1.8).

### Mutational spectrum

Retrospective genetic data from five additional patients with suspected AD HL based on the family history, clinical presentation, and/or genetic findings who had undergone standard diagnostic testing based on the suspicion of genetic HL were obtained from two medical centers in Austria, the Institute of Medical Genetics, Medical University of Vienna (Institution 1) and the Paracelsus Medical University, Salzburg (Institution 2). Clinical information and the respective analysis pipelines are contained in the Supplementary Table S4. Each variant was graded according to the ACMG/AMP guidelines. A literature search was performed to obtain previously published mutational information from Austrian AD HL patients.

## Results

### Clinical examination

Patients from 27 unrelated families from Austria were enrolled in the study, including 18 previously unpublished cases. The pedigrees and available audiograms are deposited in the Supplementary Fig. S1. The clinical presentation of previously published cases is described in the respective publications [3, 15–17].

### Variant interpretation

All causative and candidate causative variants identified in AD HL genes are summarized in Table 1. Previously published variants in *TECTA*, *COCH*, *MYO6* and *TBC1D24* are discussed in detail in the respective publications [3, 15–17]. Variants in unpublished cohort cases included known AD HL variants in *GSDME* (c.991-15_991-13del, affecting splicing [24]; AD-4), *ACTG1* (c.266C > T*,* p.Thr89Ile; AD-7)*, DIAPH1* (c.3637C > T, p.Arg1213Ter; AD-13), *NLRP3* (c.778_780delinsTGG, p.Arg260Trp; AD-16) and *WFS1* (c.2146G > A, p.Ala716Thr; in AD-26). The p.Arg1213Ter variant in *DIAPH1* (patient AD-13) was accompanied by a novel VUS in *PDE1C* (Table 1) that was considered non-causative. Segregation of the *GSDME* (AD-4) and *WFS1* (AD-26) variants was confirmed in 11 and five family members, respectively (Supplementary Fig. S1). The index patient AD-26 presented with low-frequency HL characteristic for WFS1 [10]. Novel AD HL variants included truncating alterations in the transcription factors *EYA4* (c.1658delT, p.Ile553ThrfsTer12; AD-19**)** and *POU4F3* (c.406G > T, p.Glu136Ter; in AD-20**)**, which were classified as LP based on PVS1 (established loss-of-function mechanism) and PM2 (neither is listed in gnomAD). *POU4F3* p.Glu136Ter segregated with HL in two family members (Supplementary Fig. S1). A VUS (PP3: REVEL score of 0.933, BP5) in *MYH14* (c.866 T > C, p.Ile289Thr, Table 1) in the same two affected family members was considered non-causative. A novel LP missense variant in *CEACAM16* (c.1045G > T, p.Ala349Ser, LP: PM2, PP1_Strong; AD-24) segregated in ten affected relatives. Missense variants of unknown clinical significance were identified in *ACTG1* (c.1013C > T, p.Ser338Leu, VUS: PM2, PP3; AD-11), *PTPRQ* (p.3811G > C, p.Gly1271Arg, VUS: PM2; in AD-12), and *NLRP3* (c.1904 T > C, p.Met635Thr, VUS: PM2; in AD-15), each as the sole candidate variant in the respective patient. In seven patients, no candidate variant (graded as VUS, LP, or P) was identified within coding nor splice regions, including two patients carrying likely benign (LB) variants in *TECTA* (c.3743C > T, p.Pro1248Leu, LB: BS1; in AD-8) and *WFS1* (c.1371G > T, p.Arg457Ser, LB: BS1; AD-21). Following annotation with gnomAD v3, all very rare (MAF ≤ 0.001) variants (including synonymous and intronic variants) within the covered regions of AD HL genes were extracted for patients carrying either a VUS or no candidate (Table 1). As none of those variants had a predicted effect on splicing, they were no longer considered as candidates. Variants provided from external institutions included previously described pathogenic variants in *GJB2* (c.224G > A, p.Arg75Gln; in Ext-2), *MYH9* (c.2114G > A, p.Arg705His; in Ext-4), *TMC1* (c.1714G > A, p.Asp572Asn; in Ext-5 [25]), a VUS in *WFS1* (c.437G > A, p.Arg146His, VUS: PM2; in Ext-1), and a novel LP variant in *MITF* (c.1039C > T, p.Arg347Cys, LP: PM2, PS2_moderate, PP3, PP4; in Ext-3).

**Table 1:** Causative and candidate variants identified in Austrian AD HL patients

ID	Onset (in years)		Gene	DFN locus	Hg19	Hg38	Ref	Alt	dbSNP	cDNA	Protein	MAF	CADD/ Revel	Grading
*A. Previously unpublished cases from our AD HL cohort*														
AD-4	4–6		*GSDME*	DFNA5	7: 24,746,008	7:24,706,389	GAA	–	rs727505273	NM_004403.3:c.991-15_991-13del	NP_004394.1:exon 8 skipping	–	./	P
AD-7	30		*ACTG1*	DFNA20/26	17:79,479,026	17:81,512,000	G	A	rs28999111	NM_001614.5:c.266C > T	NP_001605.1:p.Thr89Ile		24/0.783	P
AD-11	Unknown		*ACTG1*	DFNA20/26	17:79,477,831	17:81,510,805	G	A	rs1192977984	NM_001614.5:c.1013C > T	NP_001605.1:p.Ser338Leu	0.000003979 (total)	31/0.883	VUS
AD-12	14–40		*PTPRQ*	DFNA73	12:80,936,598	12:80,542,819	G	C	rs1209318209	NM_001145026.2:c.3811G > C	NP_001138498.1:p.Gly1271Arg	0.00005350 (FIN)	26.5/0.41	VUS
AD-13	Congenital		*DIAPH1*	DFNA1	5:140,903,734	5:141,524,167	G	A	rs876657776	NM_005219.5:c.3637C > T	NP_005210.3:p.Arg1213Ter	–	42/	P
			*PDE1C*	*-*	*7:31,815,322*	*7:31,775,708*	*G*	*A*	*rs201169414*	*NM_001191058.4:c.2096C* > *T*	*NP_001177987.2:p.Ser699Leu*	*0.00003730 (NFE)*	*17.77/0.2*	*VUS*
AD-15	Pre-lingual		*NLRP3*	DFNA34	1:247,588,655	1:247,425,353	T	C	-	NM_001243133.2:c.1904 T > C	NP_001230062.1:p.Met635Thr	–	19.55/3.96	VUS
AD-16	Early childhood		*NLRP3*	DFNA34	1:247,587,529	1:247,424,227	CGA	TGG		NM_001243133.2:c.778_780delinsTGG	NP_001230062.1:p.Arg260Trp	–	26.7/	P
AD-19	2–4		*EYA4*	DFNA10	6:133,844,235	6:133,523,097	T	–	–	NM_004100.5:c.1658delT	NP_004091.3:p.Ile553ThrfsTer12	–	33/	LP
AD-20	14		*POU4F3*	DFNA15	5:145,719,396	5:146,339,833	G	T	rs771471183	NM_002700.3c.406G > T	NP_002691.1:p. Glu136Ter	–	38/	LP
			*MYH14*	*DFNA4A*	*19:50,730,215*	*19:50,226,958*	*T*	*C*	*rs777836668*	*NM_001145809.2:c.866 T* > *C*	*NP_001139281.1:p.Ile289Thr*	*0.00006533 (SAS)*	*27.7/0.933*	*VUS*
AD-24	Post-lingual		*CEACAM16*	DFNA4B	19:45,211,237	19:44,707,965	G	T	rs187740201	NM_001039213.4:c.1045G > T	NP_001034302.2:p.Ala349Ser	–	23.1/0.227	LP
AD-26	4		*WFS1*	DFNA6/14/38	4:6,303,668	4:6,301,941	G	A	rs28937893	NM_006005.3:c.2146G > A	NP_005996.2:p.Ala716Thr	0.00003272 (SAS)	24.7/0.801	P
*B. Previously published cases from our AD HL cohort*														
ID	Onset (in years)		Gene	Locus	Hg19	Hg38	Ref	Alt	dbSNP	cDNA	Protein	MAF	CADD/Revel	Grading
AD-1 [17]			*TECTA*	DFNA8/12	11:121,038,785	11:121,168,076	A	G	rs121909058	NM_005422.4:c.5609A > G	NP_005413.2:p.Tyr1870Cys	–	29.3/0.826	P
AD-2 [3]	35		*COCH*	DFNA9	14:31,346,846	14:30,877,640	C	T	rs28938175	NM_004086.3:c.151C > T	NP_004077.1:p.Pro51Ser	0.000008791 (NFE)	25.4/0.873	P
AD-3 [15]	20		*TBC1D24*	DFNA65	16:2,547,068	16:2,497,067	A	C	rs1555501320	NM_001199107.2:c.919A > C	NP_001186036.1:p.Asn307His	–	24.3/0.227	LP
AD- 6 (A in [16])	4–5		*MYO6*	DFNA22	6:76,566,912	6:75,857,195	A	C	–	NM_004999.4:c.1322A > C	NP_004990.3:p.Gln441Pro	–	26.5/0.859	LP
AD-10 (E in [16])	15		*MYO6*	DFNA22	6:76,623,950	6:75,914,233	C	T	rs876657911	NM_004999.4:c.3610C > T	NP_004990.3:p.Arg1204Trp	0.00002891 (AMR)	24.6/0.863	P
AD-14 (C in [16])	4–5		*MYO6*	DFNA22	6:76,580,364	6:75,870,647	AC	–	–	NM_004999.4:c.1945_1946delAC	NP_004990.3:p.Gln650ValfsTer7	–	36/	LP
AD-22 (D in [16])	14		*GSDME*	DFNA5	7:24,745,997	7:24,706,378	C	T	–	NM_004403.3:c.991-2A > G	NP_004394.1:exon 8 skipping	–	./	P
			*MYO6*	DFNA22	6:76,538,307	6:75,828,590	C	T	rs727504567	NM_004999.4:c.238C > T	NP_004990.3:p.Arg80Ter	0.000008824 (NFE)	36/	LP
AD-23 (B in [16])	4		*MYO6*	DFNA22	6:76,576,713	6:75,866,996	C	A	–	NM_004999.4:c.1835C > A	NP_004990.3:p.Ser612Tyr	–	26.1/0.935	VUS
*AD-23 (B in *[16]*)*	4		*MYH9*	*DFNA17*	*22:36,688,079*	*22:36,292,033*	*G*	*A*	*rs727503286*	*NM_002473.6:c.4297C* > *T*	*NP_002464.1:p.Arg1433Cys*	*0.00006157 (NFE)*	*27.3/0.753*	*VUS*
AD-25 (F in [16])	20		*MYO6*	DFNA22	6:76,554,660	6:75,844,943	ACAA	–	rs749752357	NM_004999.4: c.863_866delACAA	NP_004990.3:p.Lys289ArgfsTer17	0.000008809 (NFE)	32/	P
*C. External AD HL cases provided by two additional medical institutions in Austria*														
ID	Onset (in years)		Gene	DFN locus	Hg19	Hg38	Ref	Alt	dbSNP	cDNA	Protein	MAF	CADD/ Revel	Grading
Ext-1	Pre-lingual		*WFS1*	DFNA6/14/38	4:6,290,835	4:6,289,108	G	A	rs34446752	NM_006005.3: c.437G > A	NP_005996.2: p.Arg146His	0.00009956 (AFR)	25.4/0.550	VUS
Ext-2	Pre-lingual		*GJB2*	DFNA3A	13:20,763,497	13:20,189,358	C	T	rs28931593	NM_004004.6: c.224G > A	NP_003995.2: p.Arg75Gln	–	29.6/0.99931	P
Ext-3	Pre-lingual		*MITF*	–	3:70,008,431	3:69,959,280	C	T	–	NM_001354604.2:c.1039C > T	NP_001341533.1:p.Arg347Cys	–	31/0.898	LP
Ext-4	30		*MYH9*	DFNA17	22:36,702,021	22:36,305,975	C	T	rs80338828	NM_002473.6:c.2114G > A	NP_002464.1:p.Arg705His		31/0.829	P
Ext-5 (#610 in [25])	27		*TMC1*	DFNA36	9:75,431,077	9:72,816,161	G	A	rs121908072	NM_138691.3:c.1714G > A	NP_619636.2:p.Asp572Asn	–	32/0.465	P
*D. Austrian AD HL cases gathered from literature*														
Reference		Gene		DFN locus	Hg19	Hg38	Ref	Alt	dbSNP	cDNA	Protein	MAF	CADD/Revel	Grading
[34]		GJB2		DFNA3A	13:20,763,293	13:20,189,154	C	T	rs104894401	NM_004004.6: c.428G > A	NP_003995.2: p.Arg143Gln	–	27.7/0.891	LP
[34, 35]		GJB2		DFNA3A	13:20,763,497	13:20,189,358	C	T	rs28931593	NM_004004.6: c.224G > A	NP_003995.2: p.Arg75Gln	–	29.6/0.99931	P

The table contains all causative and candidate causative genetic variants found in Austrian patients with clinically non-syndromic AD HL available to us. These include (A) previously unpublished and (B) previously published variants from our cohort, (C) previously unpublished and previously published variants provided by other institutions (Ext1-3: Institution 1; Ext-3-4: Institution 2), and (D) published variants gathered from literature. VUS that were considered non-causative are shown in Italics

### Cross-species alignment and protein structures

**Fig. 1:**
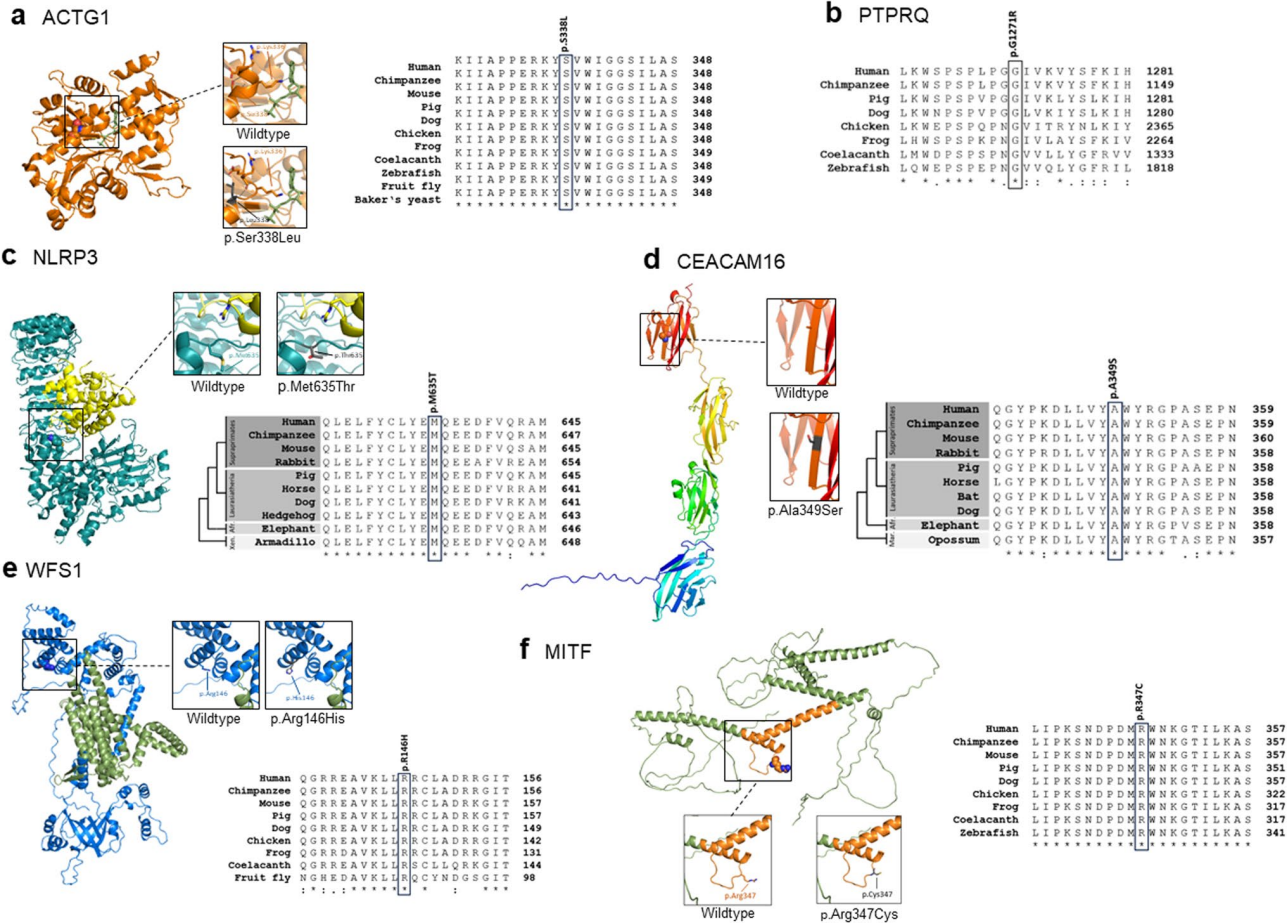
Protein models and alignments. **a** The ACTG1 serine-to-leucine substitution at position p.338 (shown as spheres) is predicted to disrupt a structurally relevant hydrogen bond, potentially altering the spatial configuration of the nucleotide-binding site. The serine residue is invariant from human to yeast. **b** No full-length PTPRQ model is available on Alphafold, and the PHYRE2 search returned no homology-based protein model for the extracellular PTPRQ domain. The mutated p.Gly1271 residue is conserved from human to zebrafish. **c** The p.Met635Thr variant (spheres) is located near the NLRP3 interaction site with NEK7, indicating that the interaction might be altered. **d** The p.Ala349Ser variant (spheres) is located on the surface of the CEACAM16 C-terminal IgV-like domain (shown in red). The variant is predicted to have no effect on the protein structure. However, due to limited knowledge of binding partners, disruption of a protein-protein interaction cannot be ruled out. The affected residue is conserved across placental mammals. **e** The Wolframin (WFS1)-mutated p.Arg146 residue is highly conserved and located in the cytosolic domain of the protein (transmembrane region shown in green). There is no predicted conformational damage from the substitution with histidine. **f** The p.Arg347Cys variant in MITF is located in the loop of the bHLH (basic helix–loop–helix) domain of the protein (orange) that is required for DNA binding. The majority of pathogenic variants linked to *MITF* HL syndromes are located in this domain [26]. The affected arginine at p.347, as well as the adjacent residues, is highly conserved

The conservation of VUS and novel missense variants in HL was assessed using cross-species alignments [15, 16]. *ACTG1* encodes the cytoplasmic gamma-actin, which is highly conserved from human to yeast. The affected serine residue at position p.338 is fully conserved across all species (Fig. 1a) and human actins (data not shown). The protein structure illustrates its location in alpha-helix α14, where it engages in hydrogen bonds with p.Glu334 and p.Arg335. Modeling of the p.Ser338Leu mutant (AD-11) predicts a possible loss of the bond with p.Arg335, which may impact the contribution of p.Lys336 to the ADP binding site (Fig. 1a). For PTPRQ, no full-length model was available. The p.Gly1271 residue mutated in family AD-12 (p.Gly1271Arg) is highly conserved (Fig. 1b). *NLRP3* (mutated in family AD-15; p.Met635Thr) belongs to the NLRP protein family that occurs in placental mammals, and the methionine at position p.635 is fully conserved across all major mammalian classes (Fig. 1c). The protein model illustrates location of the residue on the protein surface near an interaction site with the activator NEK7 (PDB-ID: 6NPY). CEACAM16 is a mammalian protein, and the peptide alignment shows full conservation of p.Ala349 across representatives from all major mammalian classes (Fig. 1d) except Xenarthra, which have no CEACAM16 orthologue. The residue p.Ala349 (AF-24; p.Ala349Ser) is located on the surface of the C-terminal IgV-like domain, and the change to serine has no predicted impact on conformation of the protein. However, due to its exposure on the surface, it might potentially be involved in interactions with other proteins. WFS1 p.Arg146 (altered in the external patient Ext-1, p.Arg146His) is evolutionary highly conserved. The residue is located in the cytosolic domain, and there is no predicted conformational impact caused by the substitution with histidine (Fig. 1e). Lastly, the mutated arginine at p.347 (external case Ext-3; p.Arg347Cys) in MITF is located in the loop of the bHLH (basic helix–loop–helix) domain involved in DNA binding. Importantly, most pathogenic variants reported for *MITF* HL syndromes affect the bHLH domain [26]. The p.Arg347 residue and adjacent residues are highly conserved across species (Fig. 1f).

## Discussion

Genetic causes underlying monogenic HL have been identified in more than a hundred genes [2], making genetic diagnoses a challenging task. Standardized variant interpretation guidelines [10–12] and a growing pool of publicly available genetic and phenotypic data continue to facilitate and improve genetic diagnoses and counseling [13, 14]. The high clinical relevance of genetic diagnoses in HL may further increase in the future when gene-therapy options become available for treatment [27]. In a continuous effort to characterize the genetic landscape of AD HL in the Austrian population, patients from 27 families were recruited at our department and screened using targeted sequencing approaches.

**Fig. 2:**
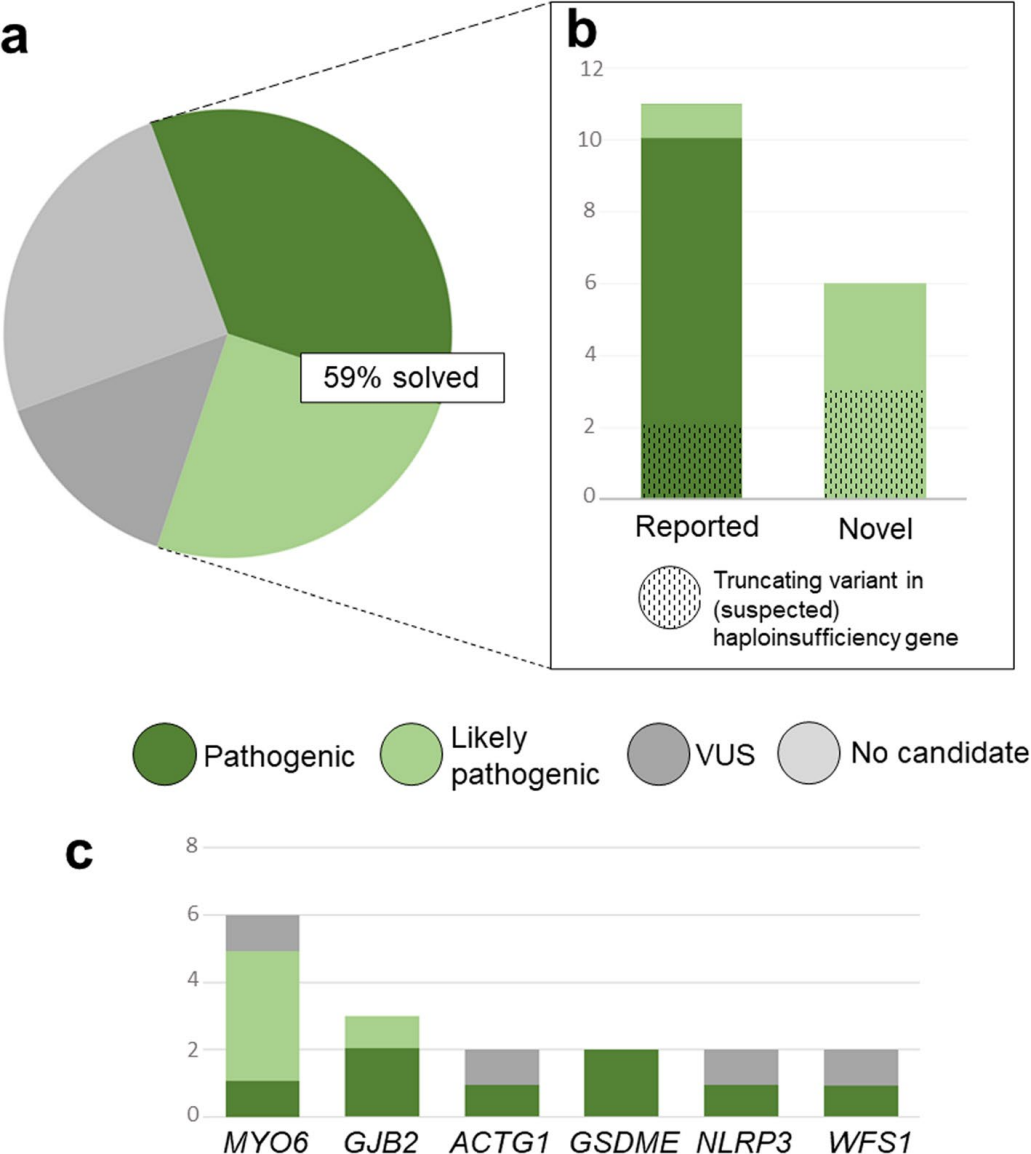
Solve rate and mutational spectrum. **a** Variants graded as pathogenic (P) or likely pathogenic (LP) were identified in 59,26% of our cohort patients (16/27). This percentage does not consider external variants or those gathered from literature. **b** Most LP and P variants identified in this cohort had previously been reported in other patients. Half of novel LP variants in our cohort were truncating variants in presumed haploinsufficiency genes. **c** Genes with more than one (candidate) causative variant, considering all Austrian patients from our cohort, external institutions and literature. As reported in [16], variants in *MYO6* were the most common cause of AD HL, though all these cases were identified in our cohort

The solve rate within the total AD HL study cohort (*n* = 27) amounted to 59,26% (16/27), considering only variants classified as LP or P as per the ACMG guidelines (Fig. 2a). This percentage is slightly higher than the diagnostic yield of other larger cohort studies with a diagnostic yield ranging from 30 to up to 50% among AD HL patients (e.g., [9, 28, 29]) and was largely accounted for by known pathogenic variants reported in literature (10/18). Moreover, loss-of-function variants in established or suspected haploinsufficiency genes (*EYA4*, *POU4F3*, and *MYO6*) were found in one third of diagnoses in the total cohort and accounted for most of the novel diagnoses (Fig. 2b), allowing for a straightforward pathogenicity assessment based on rules PVS1 and PM2 [10, 12].

Among the novel variants first published in the present study were two truncating mutations classified as LP in the transcription factor genes *EYA4* (c.1658delT, p.Ile553ThrfsTer12, in AD-19), encoding EYA Transcriptional Coactivator And Phosphatase 4, and *POU4F3* (c.406G > T, p.Glu136Ter, AD-20), encoding POU domain class 4 transcription factor 3, that have both been linked to haploinsufficiency causing post-lingual, progressive AD HL. The hearing profiles with a predominant involvement of the mid (AD-19, *EYA4,* DFNA10) and high frequencies (AD-20, *POU4F3,* DFNA15), respectively, are consistent with previous reports [30, 31]. The *POU4F3* variant in AD-20 was accompanied by a VUS (c.866 T > C, p.Ile289Thr) in *MYH14*, encoding myosin heavy chain 14, and both variants segregated with the disease in both tested family members. Since *MYH14* is typically linked to early-onset or congenital AD HL, and due to the presence of a likely alternative cause, the variant was considered non-causative. A novel rare p.Ala349Ser missense variant was identified in *CEACAM16,* encoding Carcinoembryonic antigen-related cell adhesion molecule 16. Loss-of-function variants cause post-lingual AR HL [32], and missense variants within the N-terminal IgC domain and the C-terminal IgV domain have been linked to late-onset AD HL [33]. The p.Ala349 residue affected in our patient is located on the surface of the C-terminal IgV-like domain and may contribute to an interaction site. A p.Ala349Thr variant at the same position has been classified as LB based on its high allele frequency in some populations (MAF 0.0012 (EAS), gnomAD v2.1.1). However, segregation of the variant in the large family lead to a grading of p.Ala349Ser as LP (Fig. S1).

Interestingly, one cohort patient (AD-16) whose HL had clinically been considered non-syndromic was found to carry a known pathogenic p.Arg260Trp variant in *NLRP3*, linked to cryopyrin-associated autoinflammatory syndromes. A clinical reassessment confirmed additional inflammatory manifestations consistent with AD Muckle–Wells syndrome, which includes late-onset HL. Similarly, a patient (Ext-3) who had been referred for genetic testing on the suspicion of hereditary non-syndromic HL was found to carry a heterozygous suspected de novo (paternity not confirmed) p.Arg347Cys variant in *MITF*, a gene linked to AD Waardenburg syndrome, type 2A and AD Tietz albinism-deafness syndrome, characterized by pigment loss and congenital sensorineural HL. Indeed, the patient has mild pigmentation anomalies which corroborates the potential pathogenicity of the variant. These cases demonstrate the high importance of genetic testing for precise diagnoses and prognoses.

In conclusion, our results provide insight into the mutational spectrum of AD HL in Austria and expand the current pool of genetic and clinical data in AD HL, thereby supporting other research and diagnostic laboratories in the interpretation of variants. Further, the presented data indicate a high prevalence of presumed haploinsufficiency variants in our cohort and demonstrate the importance of genetic testing for precise diagnoses in HL.

### Supplementary Information

Below is the link to the electronic supplementary material.

Supplementary file1 (PDF 1667 KB)

## Data Availability

All data relevant to the conclusions of the study are presented or referenced within the article. Additional data will be made available upon request, provided that they are not compromised by ethical restrictions.
