# Supplementary material for: Mutational spectrum in patients with dominant non-syndromic hearing loss in Austria
Source: Eur Arch Otorhinolaryngol. 2024 Feb 24;281(7):3577–86. doi: 10.1007/s00405-024-08492-5 (PMC11211180; doi:10.1007/s00405-024-08492-5)

Supplementary file

Mutational spectrum in patients with dominant non-syndromic hearing loss in Austria

Table S1. Pipelines used in the analysis of AD-7 and AD-27. The index patients of families AD-7 and AD-27 were recruited as part of our cohort and analyzed externally at a genetics institute (Institute 1) as part of a collaboration.

Patient ID	Pipeline
**AD-7**	Analyzed with the same pipeline as family A/AD-6 in [16], PMID: 33710140
**AD-27**	DNA was isolated from blood. The library was prepared with the T*ruSight One* Sequencing Panel (comprising 6713 genes), sequenced on an NextSeq 500 Illumina device, and filtered with the VarSeq software (Golden Helix, Inc.).

Table S2. Gene panel. AD HL genes targeted in the virtual gene panel.

Locus (OMIM)	Gene Symbol	HGNC	First published (PubMed ID)
DFNA20/26	ACTG1	HGNC:144	PMID: 13680526, PMID: 14684684
-	ATP2B2	HGNC:815	PMID: 30535804
DFNA44	CCDC50	HGNC:18111	PMID: 17503326
DFNA66	CD164	HGNC:1632	PMID: 26197441
DFNA4B	CEACAM16	HGNC:31948	PMID: 21368133
DFNA9	COCH	HGNC:2180	PMID: 9806553
DFNA37	COL11A1	HGNC:2186	PMID: 30245514
DFNA13	COL11A2	HGNC:2187	PMID: 10581026
DFNA40	CRYM	HGNC:2418	PMID: 12471561
DFNA1	DIAPH1	HGNC:2876	PMID: 9360932
-	DMXL2	HGNC:2938	PMID: 27657680
DFNA10	EYA4	HGNC:3522	PMID: 11159937
DFNA3A	GJB2	HGNC: 4284	PMID: 9139825
DFNA2B	GJB3	HGNC: 4285	PMID: 9843210
DFNA3B	GJB6	HGNC: 4288	PMID: 10471490
DFNA28	GRHL2	HGNC: 2799	PMID: 12393799
DFNA5	*GSDME*	HGNC:2810	PMID: 9771715
DFNA68	HOMER2	HGNC:17513	PMID: 25816005
DFNA2C	IFNLR1	HGNC:18584	PMID: 29453195
DFNA2A	KCNQ4	HGNC:6298	PMID: 10025409
DFNA69	KITLG	HGNC:6343	PMID: 26522471
DFNA7	LMX1A	HGNC:6653	PMID: 29754270
-	MAP1B	HGNC:6836	PMID: 33268592
DFNA70	MCM2	HGNC:6944	PMID: 26196677
DFNA50	MIR96	HGNC: 31648	PMID: 19363479
DFNA4A	MYH14	HGNC:23212	PMID: 15015131
DFNA17	MYH9	HGNC:7579	PMID: 11023810
-	MYO3A	HGNC:7601	PMID: 26841241
DFNA22	MYO6	HGNC:7605	PMID: 11468689
DFNA11	MYO7A	HGNC:7606	PMID: 9354784
DFNA34	NLRP3	HGNC:16400	PMID: 28847925
DFNA67	OSBPL2	HGNC:15761	PMID: 25077649, PMID: 25759012
DFNA41	P2RX2	HGNC:15459	PMID: 23345450
-	PDE1C	HGNC:8776	PMID: 29860631
-	PLS1	HGNC:9090	PMID: 31397523

DFNA15	POU4F3	HGNC:9220	PMID: 9506947
DFNA73	PTPRQ	HGNC:9679	PMID: 29309402
DFNA27	REST	HGNC:9966	PMID: 29961578
-	RIPOR2	HGNC:13872	PMID: 32631815
-	SCD5	HGNC:21088	PMID: 31972369
DFNA23	SIX1	HGNC:10887	PMID: 21700001
DFNA78	SLC12A2	HGNC:10911	PMID: 32294086
DFNA25	SLC17A8	HGNC:20151	PMID: 18674745
DFNA64	DIABLO	HGNC: 21528	PMID: 21722859
DFNA65	TBC1D24	HGNC:29203	PMID: 24729539, PMID: 24729547
DFNA8/12	TECTA	HGNC:11720	PMID: 9590290
DFNA51	TJP2	HGNC:11828	PMID: 20602916
DFNA36	TMC1	HGNC:16513	PMID: 11850618
DFNA56	TNC	HGNC:5318	PMID: 23936043
-	TRRAP	HGNC:12347	PMID: 31231791
DFNA6/14/38	WFS1	HGNC:12762	PMID: 11709537, PMID: 11709538

Table S3. Accession numbers. UniProt and Ensembl IDs of the peptide sequences used in the cross-species alignments. ACTG1 and PTPRQ peptide sequences of species from multiple mammalian and non-mammalian classes were aligned. For the mammalian proteins NLRP3 and CEACAM16, representative peptide sequences were selected from the major mammalian lineages Supraprimates (including primates (human and chimpanzee), rodents (mouse), and lagomorphs (rabbit)), Laurasiatheria (including even-toed ungulates (pig), odd-toed ungulates (horse), bats, carnivores (dog), and insectivores (hedgehog; only available for NLRP3)), Afritheria (elephant), Xenarthra (armadillo; only available for NLRP3), and marsupials (opossum, only available for CEACAM16). Afr.-Afrotheria, Mar. – Marsupialiformes, Xen. – Xenarthra.

Rank		Species	Protein					
			ACTG1	***PTPRQ***	***CEACAM16***	***NLRP3***	**WFS1**	**MITF**
Mammals	Supraprimates	*Homo sapiens*	P63261	A0A087WZU1	Q2WEN9	A0A7I2R3P8	O76024	O75030
Mammals	Supraprimates	*Pan troglodytes*	A0A2J8JGA1	A0A2I3TIG7	H2RCB2	H2Q1G8	A0A2J8JX07	A0A803K GV5
Mammals	Supraprimates	*Mus musculus*	P63260	P0C5E4	E9QA28	Q8R4B8	P56695	Q08874
Mammals	Supraprimates	*Oryctolagus cuniculus*	-	-	G1U6I2	G1SNY9	-	-
Mammals	Laurasiatheria	*Sus scrofa*	I3LVD5	A0A287ASE8	K7GQ18	H2EW08	A0A287BFZ3	A0A8D1R BX1
Mammals	Laurasiatheria	*Equus caballus*	-	-	F6RDS1	F6VLK2	-	-
Mammals	Laurasiatheria	*Myotis lucifugus*	-	-	-	G1PPW6	-	-
Mammals	Laurasiatheria	*Rhinolophus ferrumequinum*	-	-	A0A671E475	-	-	-
Mammals	Laurasiatheria	*Canis lupus familiaris*	A0A8C0Q301	A0A8C0S0W2	A0A8C0LX06	A0A8I3P020	A0A8I3S040	A0A8P0S RD5
Mammals	Laurasiatheria	*Erinaceus europaeus*	-	-	-	ENSEEUG00000 015695	-	-
Mammals	Afr.	*Loxodonta africana*	-	-	G3TLR7	G3SMV5	-	-
Mammals	Xen.	*Dasypus novemcinctus*	-	-	-	ENSDNOG0000 0005939	-	-
Mammals	Mar.	*Monodelphis domestica*	-	-	F6QGB2		-	-
Sauropsida		*Gallus Gallus*	Q5ZMQ2	A0A8V0Y8L5	-	-	A0A8V0Y4K7	A0A3Q2 U6P0
Amphibia		*Xenopus tropicalis*	Q6P378	A0A6I8SF70	-	-	A8KBD7	A4IID0
Fish		*Latimeria chalumnae*	H3A754	H3AIK6	-	-	H3ATX8	H3ATZ1
Fish		*Danio rerio*	Q7ZVI7	A0A8M9PAQ6	-	-	-	F1Q885
Inv.		*Drosophila melanogaster*	P10987	-	-	-	Q8IMZ9	-
Fungi		*Saccharomyces cerevisiae*	P60010	-	-	-	-	-

Table S4. Clinics and diagnostic screening procedures provided by external Austrian diagnostic centers.

**(a) Institution 1**	
**Patient Ext-1**	
**Clinical features**	Girl with isolated prelingual bilateral sensorineural HL. Age at the time of genetic screening: 2 y. Mother and maternal grandmother with HL and diabetes mellitus.
**Pipeline**	DNA was isolated from EDTA blood using Chemagen Magnetic Separation Module I (MSM I) by Perkin Elmer, following the manufacturer's instructions. A physical gene panel comprising *ACTG1, CCDC50, CDH23, CEACAM16, CLDN14, COCH, COL11A2, CRYM, DFNA5, DFNA50, DFNB59, DIAPH1, ESPN, ESRRB, EYA4, GIPC3, GJB2, GJB3, GJB6, GPSM2, GRHL2, GRXCR1, HGF, ILDR1, KCNQ4, LHFPL5, LOXHD1, LRTOMT, MARVELD2, MSRB3, MYH14, MYH9, MYO15A, MYO1A, MYO3A, MYO6, MYO7A, OTOA, OTOF, PCDH15, POU3F4, POU4F3, PRPS1, PTPRQ, RDX, SERPINB6, SLC17A8, SLC26A4, SLC26A5, SMAC, SMPX, STRC, TECTA, TJP2, TMC1, TMIE, TMPRSS3, TPRN, TRIOBP, USH1C, USH2A, WFS1, and WHRN was applied.* WES and analysis were performed as described for AD-7 and family A/AD-6 in [16], PMID 33710140. Mitochondrial DNA was screened using PCR and Sanger. The *WFS1* variant was validated with PCR and Sanger.
**Patient Ext-2**	
**Clinical features**	Girl with sporadic prelingual moderate-to-severe HL. Age at the time of the screening: 1 y.
**Pipeline**	The DNA was isolated from blood as described for Ext-1. The *GJB2* gene was screened using PCR and Sanger.
**Patient Ext-3**	
**Clinical features**	Girl with prelingual profound HL and mild pigmentation anomalies. Café-au-lait spots.
**Pipeline**	Clinical exome sequencing (TruSight One Expanded Sequencing Panel, Illumina) of 6713 clinically relevant genes followed by analysis of genes related to HL based on HPO terms.
**(b) Institution 2**	
**Patient Ext-4**	
**Clinical features**	Slowly progressive HL with an onset at school age. A dominant inheritance could be inferred from the family anamnesis (father with profound HL since age 49 years; three out of four children affected). In 2018 (aged 48 years), the index patient had bilateral severe-to-profound HL across all frequencies with a more pronounced loss in the high frequencies.
**Pipeline**	WES was performed as described in PMID: 31059585**.**

Continuation of Table S4b.	
**Patient Ext-5**	
**Clinical features**	The clinical features of the patient are presented in PMID: 29320412 (patient #610). In brief, the female patient developed sudden HL at the age of 27 years. The HL is symmetric, moderate-to-severe in the low frequencies and profound in the high frequencies. An autosomal dominant inheritance was deduced from the family tree. 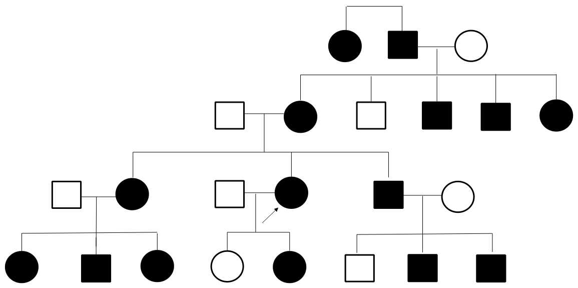
**Pipeline**	DNA was extracted from peripheral blood as described in PMID: 29320412. Prior to WES, the coding sequences of the following genes were analyzed using PCR amplification followed by Sanger sequencing; *GJB2*, *GJB6*, *SLC26A4*, *FOXI1*, *POU3F4*, *KCNJ10*, POU4F3, and *COCH,* based on the clinical finding of a bilateral enlarged vestibular aqueduct. The *SLC26A3* CEVA haplotype was assessed using a SNP assay, and multiplex PCR was used for the detection of common deletions in *GJB6.* Following negative results, WES was performed as described in PMID: 31059585**.**

Figure S1 (a-c). Pedigrees and audiograms of all previously unpublished families from our cohort. Shaded symbols: affected. Unshaded symbols: unaffected. The genotype of all family members tested by WES and/or Sanger sequencing is indicated by +/+ (homozygous wildtype) or +/- (heterozygous for the candidate causative variant). A question mark indicates that the affected status of an individual is unknown. (A) Individual IV.5 does not carry *GSDME* c.991-15_991-13del but is heterozygous for *GJB2* NM_004004.6:c.35delG.

a **AD-4** *GSDME* c.991-15_991-13del

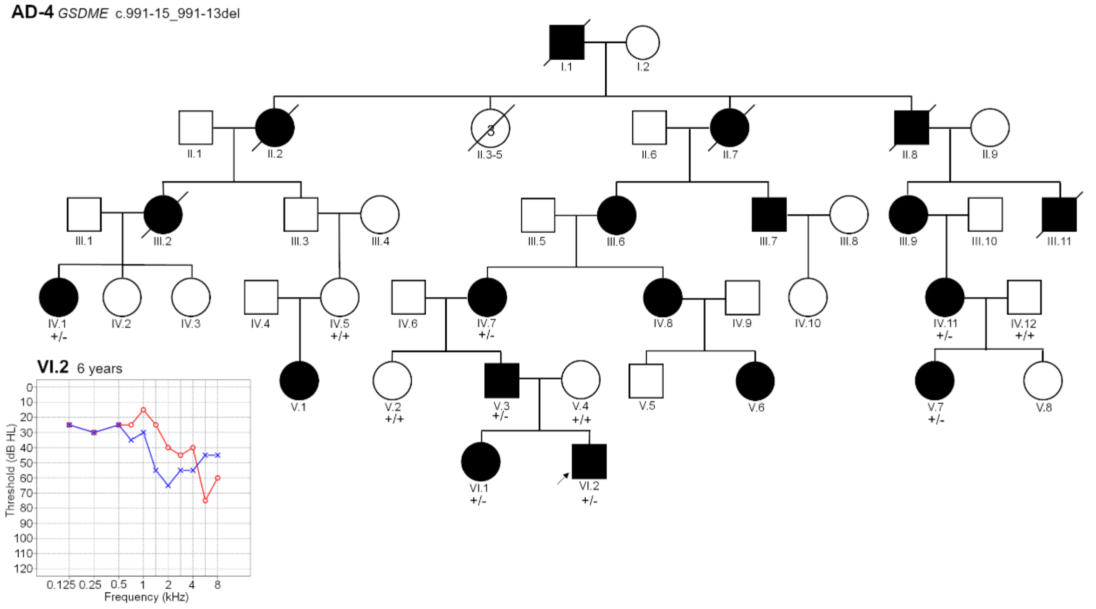

b **AD-5** No candidate

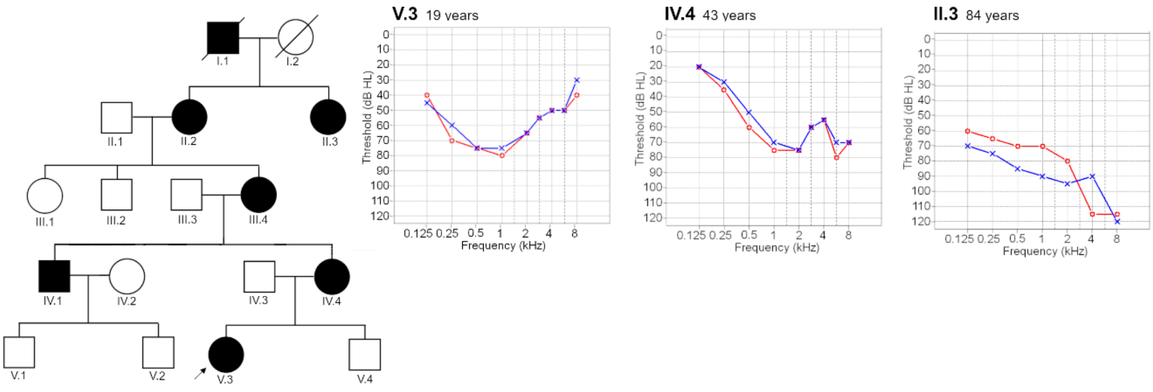

c **AD-7** *ACTG1* c.266C>T, p.Thr89Ile

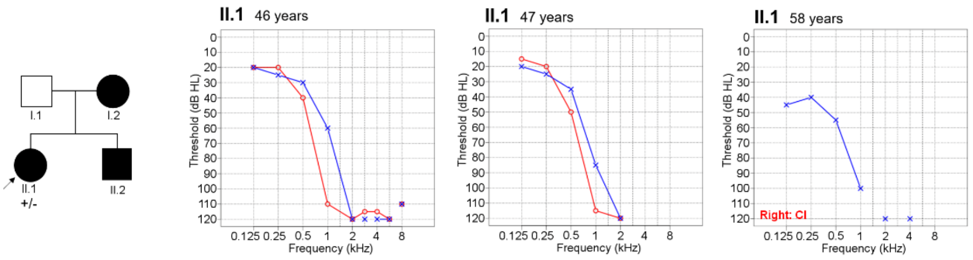

Figure S1. (d-g). Pedigrees and audiograms of all previously unpublished families.

d **AD-8** *No candidate*

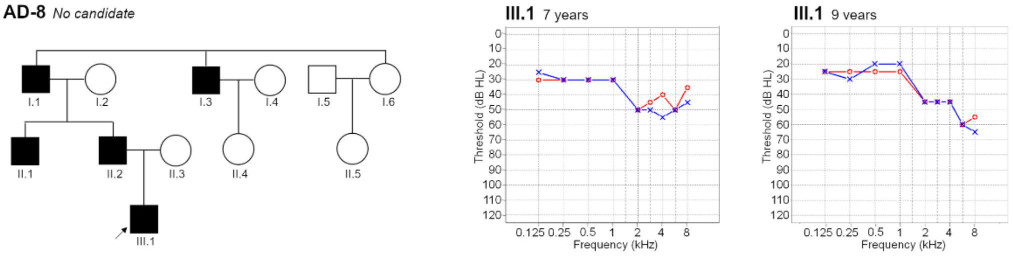

e **AD-9** *No candidate*

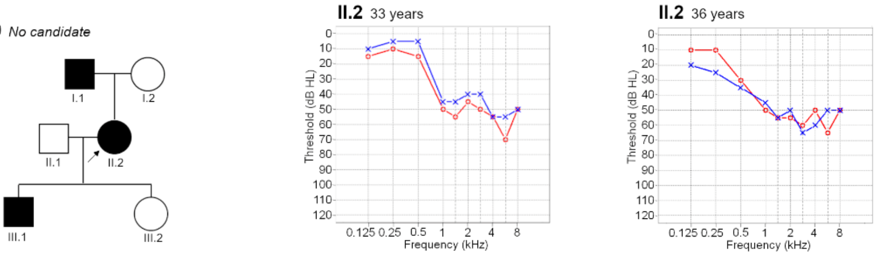

f **AD-11** *ACTG1* c.1013C>T, p.Ser338Leu

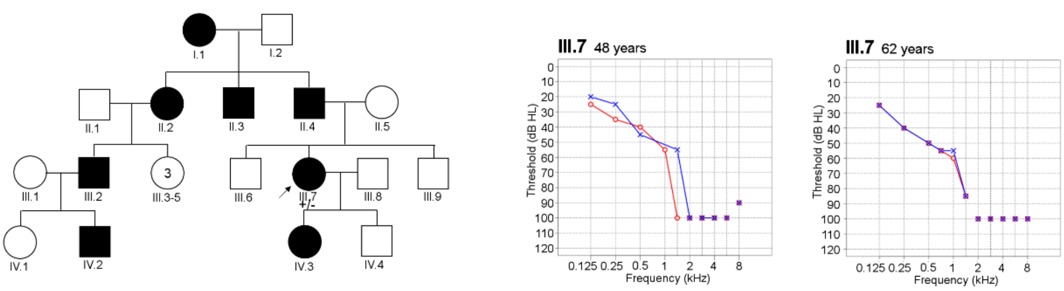

g **AD-12** *PTPRQ* p.3811G>C , p.Gly1271Arg

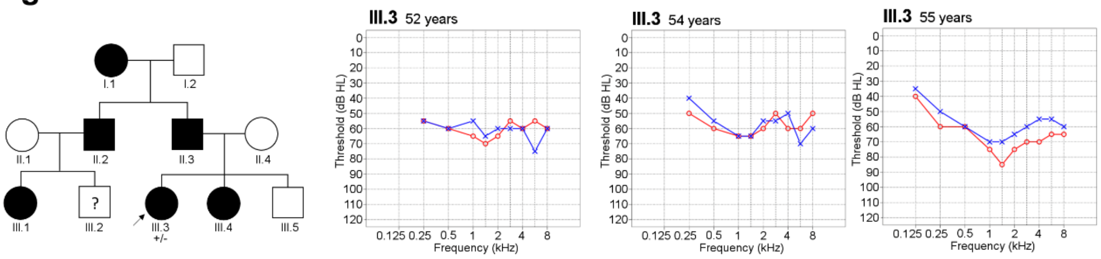

Figure S1. (h-m). Pedigrees and audiograms of all previously unpublished families. (l) No audiograms were available.

h **AD-13** *DIAPH1* c.3637C>T, p.Arg1213Ter

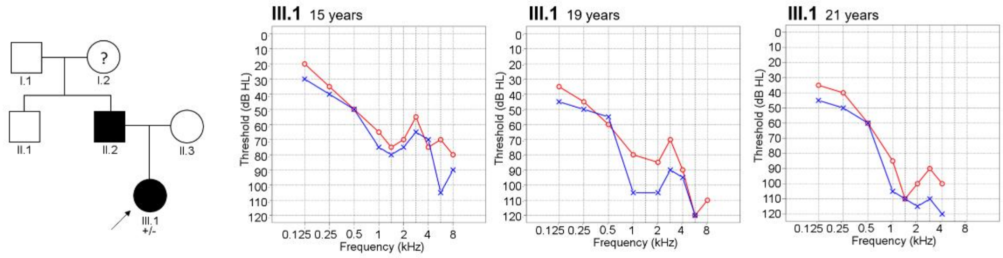

j **AD-15** *NLRP3* c.1904T>C, p.Met635Thr

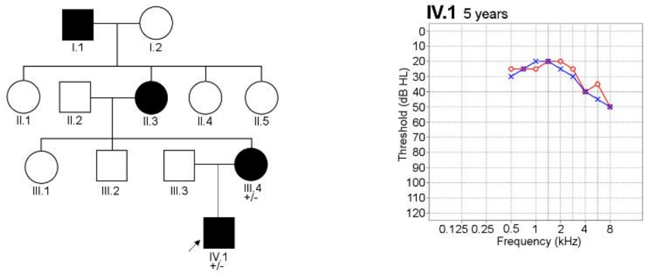

k **AD-16** *NLRP3* c.778_780delinsTGG, p.Arg260Trp

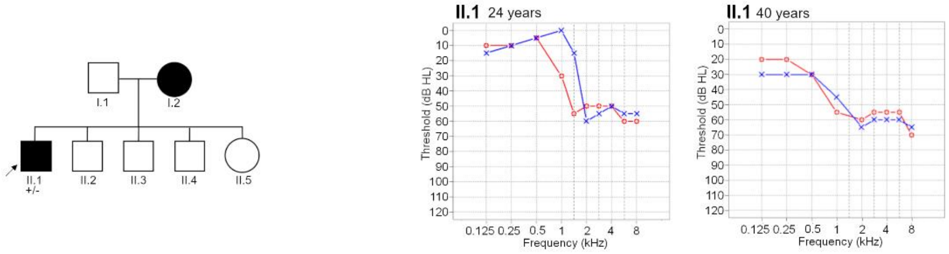

l **AD-17** No candidate

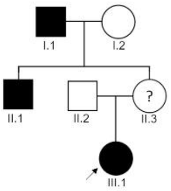

m **AD-18** No candidate

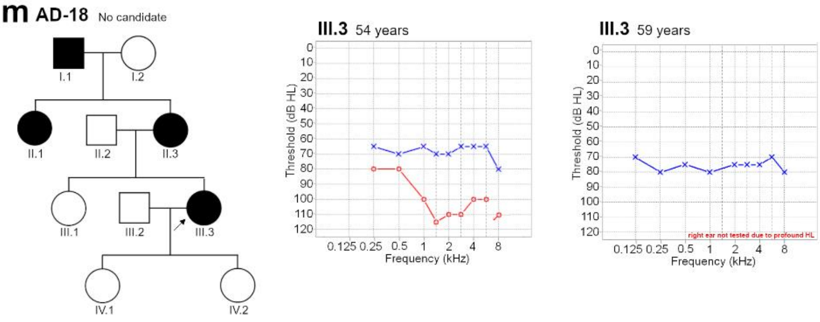

Figure S1. (n-q). Pedigrees and audiograms of all previously unpublished families. (q) Individual III.5 is affected by HL, the etiology of which was considered unclear due to a previous encephalitis.

n **AD-19** *EYA4* c.1658delT, p.Ile553ThrfsTer11

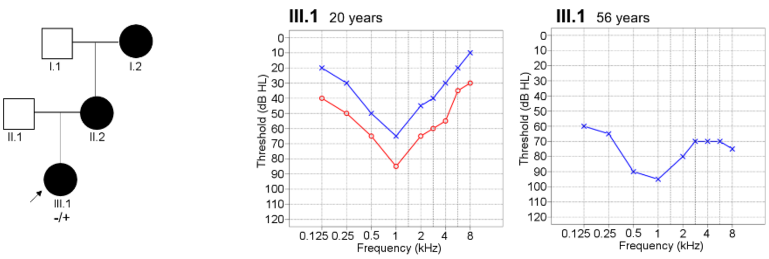

o **AD-20** *POU4F3* c.406G>T, p.Glu136Ter

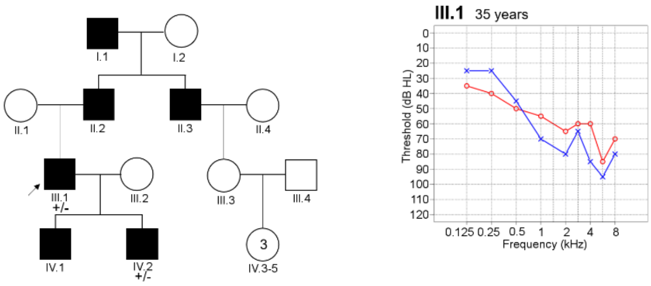

p **AD-21** No candidate

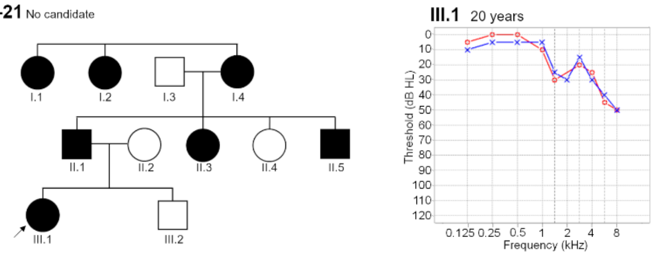

q **AD-24** *CEACAM16* c.1045G>T, p.Ala349Ser

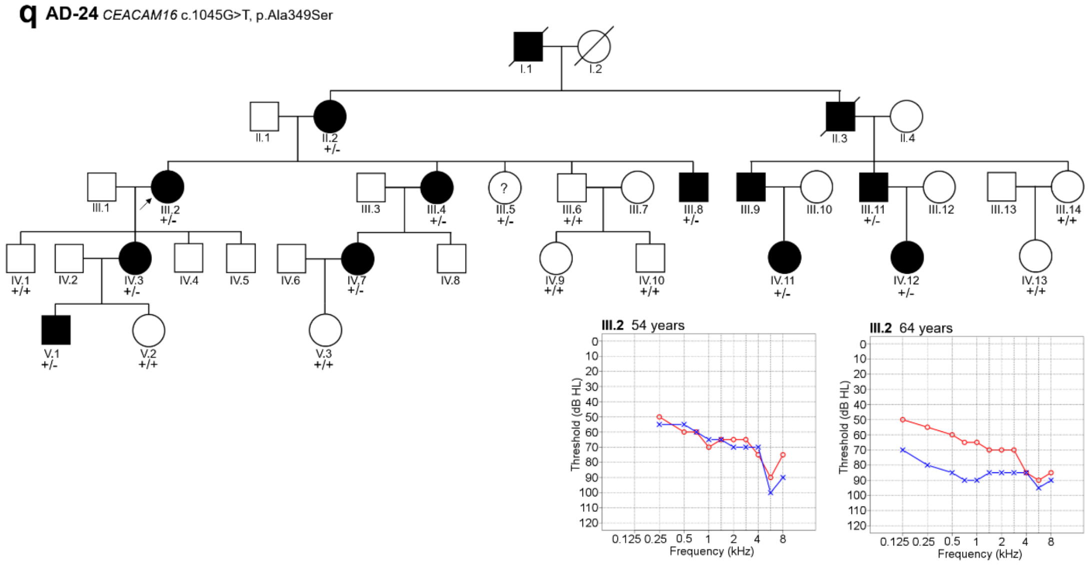

Figure S1. (r-s). Pedigrees and audiograms of all previously unpublished families.

r **AD-26** *WFS1* c.2146G>A, p.Ala716Thr

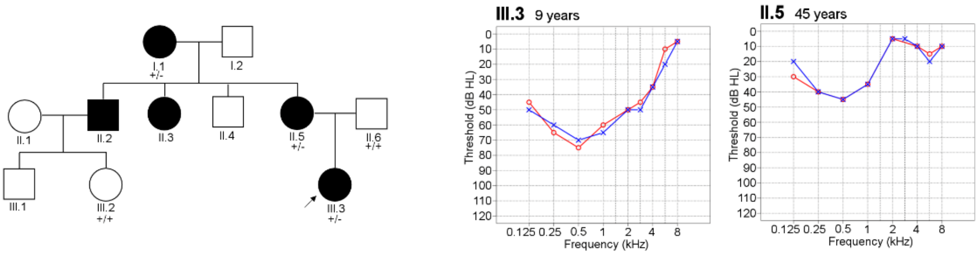

s **AD-27** No candidate

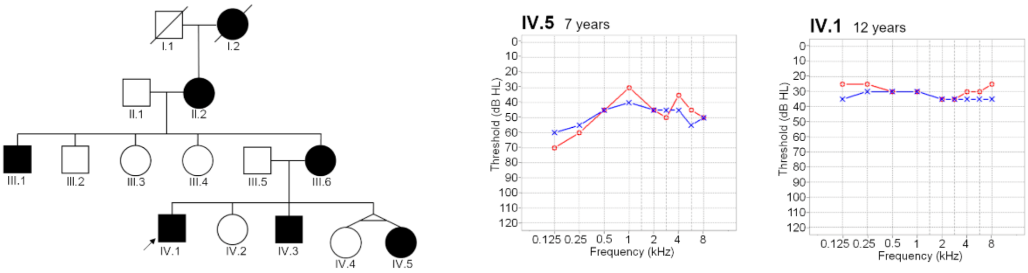

Supplement: Supplementary file 1 — Supplementary file1 (PDF 1667 KB) [file 405_2024_8492_MOESM1_ESM.pdf]
